# Psychosocial experiences of frontline nurses working in hospital-based settings during the COVID-19 pandemic - A qualitative systematic review

**DOI:** 10.1016/j.ijnsa.2021.100037

**Published:** 2021-07-17

**Authors:** Hongxuan Xu, Sigrid Stjernswärd, Stinne Glasdam

**Affiliations:** a Department of Health Sciences, Lund University, Sweden; b Health-promoting Complex Interventions, Department of Health Sciences, Faculty of Medicine, Lund University, Margaretavägen 1 B, Lund S- 222 41, Sweden; c Integrative Health Research, Department of Health Sciences, Faculty of Medicine, Lund University, Margaretavägen 1 B, Lund S- 222 41, Sweden

**Keywords:** COVID-19, Nurses, Frontline workers, Psychosocial experiences, Qualitative systematic review

## Abstract

**Background:**

Frontline nurses have been directly exposed to the SARS-CoV-2 virus and come in close contact with patients during the COVID-19 pandemic. Nurses execute tasks related to disease control and face multiple psychosocial challenges in their frontline work, potentially affecting their mental well-being and ability to satisfyingly perform their tasks.

**Objectives:**

To explore the psychosocial experiences of frontline nurses working in hospital-based settings during the COVID-19 pandemic.

**Design:**

The qualitative systematic review followed the Preferred Reporting Items for Systematic Reviews and Meta-Analyses (PRISMA) recommendations. Registered in PROSPERO (CRD42021259111).

**Data sources:**

Literature searches were performed through PubMed, CINAHL, and the WHO COVID-19 database. Inclusion criteria were: All types of nurses having direct contact with or taking care of patients; Primary, secondary, and tertiary health-care services admitting and treating COVID-19 patients; Experiences, perceptions, feelings, views in psychosocial aspects from the identified population group; Qualitative studies; Mixed methods studies; Language in English; Published date 2019–2021. Exclusion criteria were: Commentaries; Reviews; Discussion papers; Quantitative studies; Language other than English; Published in 2018 or earlier; Studies without an ethical approval and ethical statement.

**Review methods:**

The studies were screened and selected based on the inclusion and exclusion criteria. Quality appraisal was conducted according to the Critical Appraisal Skills Program qualitative study checklist. Data was extracted from included studies and a thematic synthesis was made.

**Results:**

A total of 28 studies were included in the review. The experiences of 1141 nurses from 12 countries were synthesised. Three themes were constructed: ‘Nurses’ emotional, mental and physical reactions to COVID-19′, ‘Internally and externally supported coping strategies’, and ‘A call for future help and support’.

**Conclusion:**

Nurses working frontline during the COVID-19 pandemic have experienced psychological, social, and emotional distress in coping with work demands, social relationships, and their personal life. The results pointed to a need for increased psychological and social support for frontline nurses to cope with stress and maintain mental well-being, which may subsequently affect nursing care outcomes.

**What is already known about the topic:**

• Nurses have close contact with the COVID-19 infected patients.

• Nurses are placed in unpredictable and high-risk situations entailing increased probabilities of distress.

**What this paper adds:**

• Frontline nurses experienced fear, anxiety, and psychological stress due to the perceived risk of infection, uncertainty, and concerns about family members.

• The unfamiliarity of work conditions and psychological unpreparedness were main occupational stressors.

• External support enhanced nurses’ coping abilities during the pandemic.

Alt-text: Unlabelled box

## 1 Introduction

The World Health organisation (WHO) declared the outbreak of the SARS-CoV-2 virus that causes coronavirus disease (COVID-19) to be a Public Health Emergency of International Concern and to be characterised as a pandemic (World Health Organization 2020). Common symptoms of COVID-19 are fever, dry cough, and shortness of breath (World Health Organization 2020). Besides, the SARS-CoV-2 virus attacks the lungs, and can infect the heart, kidneys, liver, brain, and intestines (World Health Organization 2020). The virus is mainly spread through saliva droplets or discharged from the nose when an infected person coughs or sneezes (World Health Organization 2020) and by airborne transmission through aerosols (Klompas et al., 2020). Healthcare professionals are the main personnel involved in screening and treatment on the frontline of the COVID-19 pandemic (Spoorthy et al., 2020). Frontline healthcare professionals are here defined in line with Nguyen et al. (Nguyen et al., 2020) as individuals who reported direct patient contact.

As the major component of the hospital workforce in relation to the COVID-19 pandemic, nurses care for all types of patients and have most contact with COVID-19 infected patients (Schroeder et al., 2020, Gesesew et al., 2021). Frontline nurses are directly exposed to the SARS-CoV-2 virus and come in close contact with patients in care situations, undertaking most of the tasks related to the control of the COVID-19 pandemic (Hu et al., 2020). As such, nurses themselves are at high risk of being infected with COVID-19 (Fernandez et al., 2020, Liu et al., 2012). The COVID-19 pandemic has led to an unforeseen shift in nursing practice to meet the sudden increase in demand for pandemic-related care (Schroeder et al., 2020). Personal protective equipment (PPE) creates barriers to the efficacy of nurse-patient communication and physical contact, including restricted contact between patients and their family members (COVID-19: Changing the Face of the Nurse-Patient Relationship 2020). It means that frontline nurses are unable to provide adequate services to patients in the way they were taught and expected to, resulting in increased pressure for the nurses and dissatisfaction with their work (COVID-19: Changing the Face of the Nurse-Patient Relationship 2020). Studies show that nurses experience mental and physical stress at work, with subsequent negative health effects, when facing excessive workload, ambiguity in roles, and interpersonal conflict in their general work (Pisanti et al., 2015, Pisanti et al., 2016, Giorgi et al., 2016). Therefore, nurses working during the COVID-19 pandemic are both exposed to occupational hazards and psychosocial pressures at the same time (Pisanti et al., 2015).

Nurses are placed in unpredictable and high-risk situations which entail increased probabilities of physical, mental, and emotional distress (Lai et al., 2020, Huang et al., 2020, Rodríguez and Sánchez, 2020), while impacting the quality and safety of the care they deliver (World Health Organization 2020). Compared with the other health-care professionals, nurses had a higher prevalence of anxiety, depression, and post-traumatic stress disorder (PTSD) during and after pandemics (Martikainen et al., 2002, Maunder et al., 2004, Maunder et al., 2006, Chong et al., 2004, Bai et al., 2004, Verma et al., 2004, Barello et al., 2020, Grace et al., 2005). The WHO points out that healthcare professionals are facing multiple psychosocial hazards during the COVID-19 pandemic, such as long working hours and high workload, which can lead to fatigue, occupational burnout, increased psychological distress and/or decreased mental health (World Health Organization 2020). Psychosocial factors refer to the influences of social characteristics on psychological and mental health, as well as behaviours of a person (Martikainen et al., 2002, Macleod and Davey, 2003). Psychosocial factors consist of multidimensional domains encompassing mood status, cognitive behavioural responses, and social factors (Suzuki and Takei, 2013). Protecting the nurses’ mental well-being by providing adequate psychosocial support during the COVID-19 pandemic has been identified as essential to ensure the long-term capacity of the health workforce (World Health Organization 2020).

Due to different responses to the pandemic, many countries and regions have repurposed and restructured hospitals to distribute the medical burden and prevent bed shortages (Her, 2020). The challenges and stress during the pandemic faced by nurses are significant (Cox, 2020, Kang et al., 2020), and varied task distributions and levels of experience among nurses may lead to various frontline work experiences. Understanding the psychosocial experiences of frontline nurses is essential to ensure that nurses are adequately supported and that the workforce and delivery of high-quality care during the period of increased health care need is maintained (Fernandez et al., 2020). Therefore, the aim of current study was to synthesize research literature about the psychosocial experiences of frontline nurses working in hospital-based settings during the COVID-19 pandemic.

## 2 Methods

A qualitative systematic review was undertaken to synthesize the findings from qualitative primary studies to provide in-depth insights into frontline nurses’ psychosocial experiences. Systematic reviews are regarded as the standard of evidence-based practice, and are increasingly used for policy decisions and research directions (Aromataris and Riitano, 2014). This article followed the Preferred Reporting Items for Systematic Reviews and Meta-Analyses (PRISMA) recommendations (Moher et al., 2009, Liberati et al., 2009), see supplementary file 1 and 2. The review protocol is registered in PROSPERO (CRD42021259111).

### 2.1 Eligibility criteria

Study characteristics were identified by Population, Exposure, Outcomes (PEO) representing a framework to design research questions for qualitative studies and reviews, and to develop search strategies (Butler et al., 2016). P: frontline nurses that have been in contact with or taken care of patients during work in the COVID-19 pandemic, E: working during the COVID-19 pandemic, O: psychosocial experiences of nurses working during the COVID-19 pandemic. Psychosocial experience in this study is defined as the subjective experiences, perspectives, feelings, and views of the influences on mood status, cognitive behavioral responses, and social factors of a person (Suzuki and Takei, 2013).

Different keywords to be used were listed in Table 1, as well as types of studies to be included in the review. The population included all types of nurses involved in caring for patients, because the nursing role can vary due to needs associated with a pandemic (Hovan, 2020). The exposure included primary, secondary, and tertiary health-care settings that admit and treat COVID-19 patients. Depending on the country, its coping strategies, and its various circumstances or stages during the pandemic, the COVID-19 designated locations may differ. In view of the study's aim, only COVID-19 designated wards or primary health-care settings admitting and treating COVID-19 patients were included (World Health Organization, 2020). Given the special nature of nursing homes and any other health-care facilities that were non-designated for admitting and treating COVID-19 patients, studies conducted in those settings were excluded. Further, studies without an ethical approval and/or ethical statement were excluded. Outcomes included experiences, perceptions, feelings, views of psychosocial issues from the identified population group during the COVID-19 pandemic.

**Table 1:** Inclusion and exclusion criteria.

	PEO	Inclusion criteria	Exclusion criteria
Population	Frontline nurses that have been in contact with or taken care of patients during work in the COVID-19 pandemic.	All types of nurses involved, such as Registered Nurse (RN), Licensed Practical Nurse (LPN), Nurse Practitioner (NP), Specialized Nurses.Having direct contact with patients.Taking care of patients.	Nursing assistants.Nursing students.Informal caregiversNurses’ professional activities do not include taking care of patients. The entire group of HCWs, without specified data for nurses.
Exposure	Working during the COVID-19 pandemic	Primary, secondary, and tertiary health-care services admitting and treating COVID-19 patients. COVID-19 designated hospitals.Infectious disease hospitals.COVID-19 field hospitals.	Health care services for case investigation, national laboratories, early investigation protocols, and community engagement (World Health Organization 2020).Nursing homes and other health-care facilities that are not designated to admit and treat COVID-19 patients.
Outcome	Psychosocial experiences of nurses working during the COVID-19 pandemic	Experiences, perceptions, feelings, views in psychosocial aspects from the identified population group.	The psychosocial experience was not due to the COVID-19 pandemic.The experience or perspective was not relevant to psychosocial aspects.
Type of study		Action Research;Grounded theory;Ethnonursing Research;Ethnological Research;Ethnographic Research;Naturalistic Inquiry;Phenomenological Research; Narrative research;Mixed methods with elements of qualitative analysis.	Commentaires;Reviews;Discussion papers;Quantitative studies.
Others		Language in English; Published date 2019–2021;Available full-text articles.	Language other than English;Published in 2018 or earlier;Studies without an ethical approval and ethical statement.

### 2.2 Literature search

The search was conducted in PubMed, CINAHL, and the WHO COVID-19 database on December 8, 2020 (Global research on coronavirus disease (COVID-19) 2020). A systematic search was conducted to identify all peer-reviewed and original empirical qualitative studies that answer the research aim (Bettany-Saltikov and McSherry, 2016). In each of the three selected databases, the search strategy consisted of a building block search carried out according to the PEO framework. The citation pearl search was conducted within Web of Science (WOS) to assess the importance and relevance of included studies, as well as to ensure that all relevant studies were included. WOS brings together all cited references for the citation search and contains citation indexes from each reference list. The years 2019–2020 were chosen to make sure that we did not miss any literature as the virus was first identified in humans in December 2019 in China, despite the fact that the outbreak was officially made public only in January 2020 (World Health Organization 2020). Table 2 shows the full electronic search strategy used to identify studies, including all the search terms and limits for all three databases.

**Table 2:** The full electronic search strategy for all three databases.

Database	Search terms	Filters	Outcomes
PubMed	(COVID-19 OR corona virus OR COVID OR covid-19 OR covid pandemic OR Coronavirus OR SARS-CoV-2 OR coronavirus disease OR 2019-nCoV) AND (nurses OR nursing staffs OR nurse OR nursing staff OR auxiliary nurses OR Nursing Assistants OR ("Nursing Assistants"[M*esh*]) OR ("Emergency Nursing"[M*esh*]) OR ("Nursing"[M*esh*]) OR ("Nursing Care"[M*esh*]) OR ("Nursing Staff, Hospital"[M*esh*]) OR ("Nursing Staff"[M*esh*]) OR ("Nurses"[M*esh*])) AND (("Psychology"[M*esh*]) OR perceived stress OR psychological responses OR psychosocial functioning OR mood status OR psychosocial aspects OR psychosocial process OR psychosocial experiences OR psychosocial OR psychosocial impacts OR psychosocial changes OR social aspects OR ("Social Environment"[M*esh*])) OR ("Emotions"[M*esh*]) OR ("psychology" [Subheading]) OR ("Emotions"[M*esh*]) OR("Psychosocial Deprivation"[M*esh*]) OR ("Emotional Adjustment"[M*esh*]) OR ("Emotional Regulation"[M*esh*]) OR ("Depression"[M*esh*]) OR ("Social Desirability"[M*esh*]) OR psychological, social behavior))	Review/Scientific Integrity Review/Systematic Review, quantitative study, English, Publication Date 2019–2020	428
**CINAHL**	((MH "COVID-19″) OR COVID-19 OR corona virus OR COVID OR covid-19 OR covid pandemic OR Coronavirus OR SARS-CoV-2 OR coronavirus disease OR 2019-nCoV) AND ((MH "Registered Nurses") OR (MH "Nursing Assistants") OR (MH "Emergency Nursing+") OR (MH "Nursing Care+") OR (MH "Nursing Staff, Hospital") OR (MH "Nurses+") OR nurses OR nursing staffs OR nurse OR nursing staff OR auxiliary nurses OR Nursing Assistants ) AND (perceived stress OR psychological responses OR psychosocial functioning OR mood status OR psychosocial aspects OR psychosocial process OR psychosocial experiences OR psychosocial OR psychosocial impacts OR psychosocial changes OR social aspects OR (MH "Emotions+") OR (MH "Psychosocial Health (Iowa NOC)+") OR (MH "Psychological Distress") OR (MH "Social Behavior+") OR (MH "Social Behavior Disorders+") OR (MH "Psychosocial Aspects of Illness+") OR (MH "Psychology, Social+") OR (MH "Psychosocial Deprivation") OR (MH "Social Isolation+") OR (MH "Social Values+") OR (MH "Public Relations+") OR (MH "Stress Disorders, Post-Traumatic+") OR (MH "Psychosocial Adjustment: Life Change (Iowa NOC)") OR (MH "Psychosocial Adaptation (Iowa NOC)+"))	Peer Review, qualitative study, English, Publication Date 2019–2020	363
WHO COVID-19 database	(tw:(psychosocial functioning) OR (tw:(Psychosocial Factors)) OR (tw:(Psychosocial experiences)) OR (tw:(Psychosocial feeling)) OR (tw:(psychosocial process)) OR (tw:(perceived stress)) OR (tw:(psychological responses)) OR (tw:(psychosocial impacts)) OR (tw:(psychosocial changes)) OR (tw:(social aspects)) OR (tw:(mood status )) OR (tw:(Psychosocial Outcomes)) OR (tw:(Psychosocial Readjustment)) OR (tw:(Emotional Control)) OR (tw:(Emotional Adjustment)) OR (tw:(Emotional and Behavioral Disorders)) OR (tw:(Psychological Development)) OR (tw:(Cognitive Development)) OR (tw:(Emotional Development)) OR (tw:(Psychosocial Development)) OR (tw:(Social Acceptance)) OR (tw:(Psychological Stress)) OR (tw:(Social and Interpersonal Measures)) OR (tw:(Social Adjustment)) OR (tw:(Social Behavior)) OR (tw:(Social Functioning)) OR (tw:(Occupational Stress)) OR (tw:(Stress Management)) OR (tw:(Psychosocial Health)) OR (tw:(Psychosocial Aspects of Illness)) OR (tw:(Stress Disorders)) OR (tw:(Psychosocial Adaptation)) OR (tw:(Emotions))) AND (tw:(Nurses $) OR (tw:(nursing staffs)) OR (tw:(nurses)) OR (tw:(Nursing Assistants)) OR (tw:(Emergency Nursing)) OR (tw:(registered nurse)) OR (tw:(licensed practical nurse)) OR (tw:(nurse practitioner)) OR (tw:(specialized nurses)) OR (tw:( nursing staffs)))	Peer Review, qualitative study, English, Publication Date 2019–2020	106

### 2.3 Study selection

**Fig. 1:**
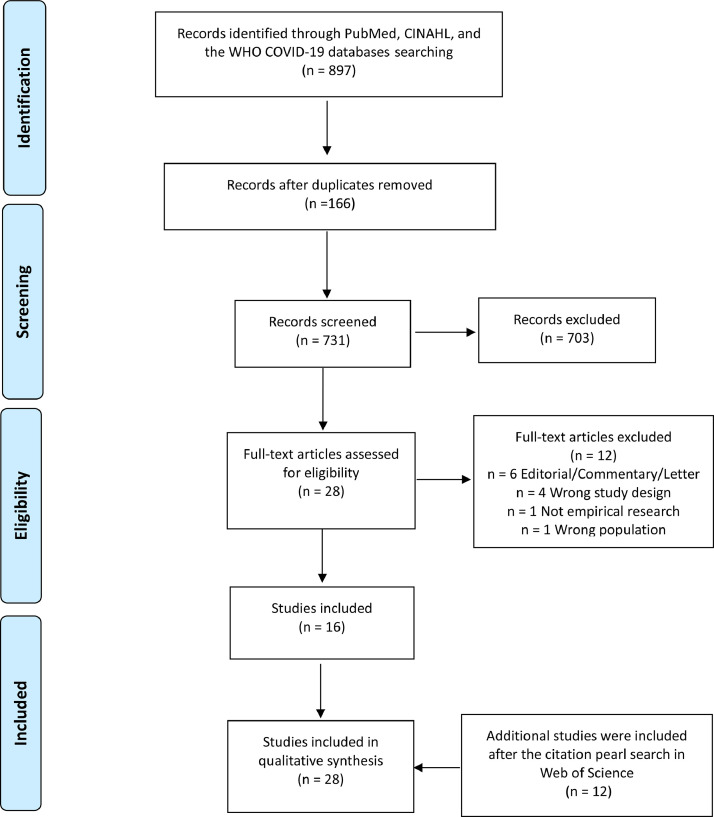
PRISMA flow diagram.

The initial search retrieved 897 studies, which were transferred to Covidence software for the following screening process (Veritas Health Innovation Covidence Systematic Review Software [computer program], 2020). The entire study selection process was conducted collaboratively by two authors (HX and SG). In case of disagreement in the screening or full-text review process, the two authors discussed until an agreement was reached. According to the inclusion and exclusion criteria, 16 eligible studies were combined through a final step with 12 additional studies that were retrieved through a citation pearl search. The additional studies from the citation pearl search were included through the joint decisions of all the authors. Finally, 28 studies that met the inclusion criteria were assessed for quality and included for synthesis (Arnetz et al., 2020, Eftekhar et al., 2020, Fan et al., 2020, N Galehdar et al., 2020, Gao et al., 2020, Góes et al., 2020, Hou et al., 2020, Jia et al., 2021, Kackin et al., 2020, Kalateh et al., 2020, Q Liu et al., 2020, YE Liu et al., 2020, Sheng et al., 2020, Sun et al., 2020, Tan et al., 2020, Zhang et al., 2020, Deliktas et al., 2021, Muz and Erdoğan, 2021, Coşkun and Günay, 2021, N Galehdar et al., 2020, Sethi et al., 2020, Fernández-Castillo et al., 2021, Ohta et al., 2020, Lee and Lee, 2020, Goh et al., 2020, Bennett et al., 2020, Vindrola-Padros et al., 2020, Okediran et al., 2020). The study selection process is presented in a PRISMA flow diagram (Fig. 1). The 28 included studies are marked with an asterisk * in the references.

### 2.4 Appraisal of study quality

The Critical Appraisal Skills Program (CASP) qualitative study checklist was used to appraise the quality of included studies (Critical Appraisal Skills Programme 2018). It consists of ten questions that assess a study's aim, methodology and design, recruitment strategy, data collection, data analysis, findings, and research value, see Table 3. No studies were excluded in this process.

**Table 3:** CASP Study appraisal form.

Authors	Section A: Are the results valid?						Section B: What are the results?			Section C: Will the results help locally	Outcomes
	1. Was there a clear statement of the aims of the research?	2. Is a qualitative methodology appropriate?	3. Was the research design appropriate to address the aims of the research?	4. Was the recruitment strategy appropriate to the aims of the research?	5. Was the data collected in a way that addressed the research issue?	6. Has the relationship between researcher and participants been adequately considered?	7. Have ethical issues been taken into consideration?	8. Was the data analysis sufficiently rigorous?	9. Is there a clear statement of findings?	10. How valuable is the research?	
Arnetz et al., 2020	Yes	Yes	Yes	Yes	Yes	Yes	Yes	Yes	Yes	Yes	10/10
Eftekhar et al., 2020	Yes	Yes	Yes	Yes	Yes	Yes	Yes	Yes	Yes	Yes	10/10
Fan et al., 2020	Yes	Yes	Yes	Yes	Yes	No	Yes	Yes	Yes	Yes	9/10
N Galehdar et al., 2020	Yes	Yes	Yes	No	Yes	Yes	Yes	Yes	Yes	Yes	9/10
Gao et al., 2020	Yes	Yes	Yes	Yes	Yes	No	Yes	Yes	Yes	Yes	9/10
Góes et al., 2020	Yes	Yes	Yes	Yes	Yes	No	Yes	Yes	Yes	Yes	9/10
Hou et al., 2020	Yes	Yes	Yes	Yes	Yes	Yes	Yes	Yes	Yes	Yes	10/10
Jia et al., 2021	Yes	Yes	Yes	Yes	Yes	No	Yes	Yes	Yes	Yes	9/10
Kackin et al., 2020	Yes	Yes	Yes	Yes	Yes	Yes	Yes	Yes	Yes	Yes	10/10
Kalateh et al., 2020	Yes	Yes	Yes	Yes	Yes	No	Yes	Yes	Yes	Yes	9/10
Q Liu et al., 2020	Yes	Yes	Yes	Yes	Yes	No	Yes	Yes	Yes	Yes	9/10
YE Liu et al., 2020	Yes	Yes	Yes	Yes	Yes	No	Yes	Yes	Yes	Yes	9/10
Sheng et al., 2020	Yes	Yes	Yes	Yes	Yes	No	Yes	Yes	Yes	Yes	9/10
Sun et al., 2020	Yes	Yes	Yes	Yes	Yes	Yes	Yes	No	Yes	Yes	9/10
Tan et al., 2020	Yes	Yes	Yes	Yes	Yes	Yes	Yes	Yes	Yes	Yes	10/10
Zhang et al., 2020	Yes	Yes	Yes	Yes	Yes	No	Yes	Yes	Yes	Yes	9/10
Deliktas et al., 2021	Yes	Yes	Yes	Yes	Yes	Yes	Yes	Yes	Yes	Yes	10/10
Muz and Erdoğan, 2021	Yes	Yes	Yes	Yes	Yes	No	Yes	Yes	Yes	Yes	9/10
Coşkun and Günay, 2021	Yes	Yes	Yes	Yes	Yes	No	Yes	Yes	Yes	Yes	9/10
N Galehdar et al., 2020	Yes	Yes	Yes	Yes	Yes	No	Yes	Yes	Yes	Yes	9/10
Sethi et al., 2020	Yes	Yes	Yes	Yes	Yes	Yes	Yes	Cannot tell	Yes	Yes	9/10
Fernández-Castillo et al., 2021	Yes	Yes	Yes	Yes	Yes	No	Yes	Yes	Yes	Yes	9/10
Ohta et al., 2020	Yes	Yes	Yes	No	Yes	Yes	Yes	Yes	Yes	Yes	9/10
Lee and Lee, 2020	Yes	Yes	Yes	Yes	Yes	No	Yes	Yes	Yes	Yes	9/10
Goh et al., 2020	Yes	Yes	Yes	Yes	Yes	No	Yes	Yes	Yes	Yes	9/10
Bennett et al., 2020	Yes	Yes	Yes	Yes	Yes	Yes	Yes	Yes	Yes	Yes	10/10
Vindrola-Padros et al., 2020	Yes	Yes	Yes	Yes	Yes	Yes	Yes	Yes	Yes	Yes	10/10
Okediran et al., 2020	Yes	Yes	Yes	Yes	Yes	No	Yes	Yes	Yes	Yes	9/10

### 2.5 Data extraction

Data extraction was performed to highlight the qualitative data of primary studies that was relevant to the review aim (Bettany-Saltikov and McSherry, 2016, Noyes and Lewin, 2011). Qualitative data referred to non-numerical or non-measurable information that captured a person's opinions or described the person's lived experiences (Tuckerman et al., 2020). The extracted data items were informed by the review's aim, including author(s), year, nation of publication, and setting(s); study aim(s) or research question(s); study design and theoretical framework; sampling strategy and participants characteristics; data collection methods; data analysis methods; ethical issues; and major findings, see Table 4.

**Table 4:** Study characteristics .

Author(s) YearCountrySetting(s)	Study aim(s) /research question(s)	Study design; Theoretical framework	Sampling strategy; Participants’ characteristics	Data collection methods	Data analysis methods	Major findings
(Arnetz et al., 2020)USAInpatient/hospital/ outpatient settings	To explore perceptions of the most salient sources of stress in the early stages of the coronavirus pandemic in a sample of U.S. nurses.	Cross-sectional survey study	• Snowball recruitment technique• Nurses: *N* = 455• 429 females, 26 males. • Age range: younger than 35 - 65 or older• Mean age: NR• Work experience range: less than 5 years - 10 years or more• Mean work experience: NR	Open-ended question in a survey	Qualitative content analysis	• The fear of the self being exposed to COVID-19 and becoming ill. • The fear of passing virus onto others. • The infection and death of others, work-related problems, stressors related to PPE/Supplies, dealing with unknowns, and family/community opinions. • Restrictions associated with the pandemic and feelings of inadequacy/helplessness regarding patients and their treatment.
(Eftekhar et al., 2020)IranHospitals	To undertake an in-depth exploration of the experiences and the mental health consequences of health-care staff working during the COVID-19 crisis.	Qualitative study	• Maximum variation sampling• Nurses: *N* = 36• Age range: NR• Mean age: NR• Work experience range: NR• Mean work experience: NR	Semi-structured, in-depth interviews by using telephone and video calls	Thematic analysis	• High levels of stress, fear, and anxiety among healthcare providers in the early phases of the pandemic. • The sense of helplessness, hopelessness and becoming powerless was prevalent among them.
(Fan et al., 2020)ChinaHospitals	To collect the experiences and views of transdisciplinary nurses at the forefront of the COVID-19 outbreak and to evaluate their psychological stresses.	Qualitative study	• Purposeful sampling method• Nurses: *N* = 44 • 38 females, 6 males• Age range: 20–40 or older• Mean age: NR• Work experience range: 1–15 or more• Mean work experience: NR	Semi-structured and face-to-face interviews	Thematic Analysis method	• Higher perceived stress levels and less perceived social support were detected in the transdisciplinary nurse (TN).• Ambiguous roles. • The transition of operating modes. • Unfamiliar work contents, the work environment and intensity, and the reversal of daily schedules.• Psychological problems. • Sense of powerlessness, incomprehension of parents, concern for family members and long-term isolation.
(N Galehdar et al., 2020)IranHospitals	To explore nurses’ experiences of psychological distress during care of patients with COVID-19.	Qualitative study	• Purposeful sampling method• Nurses: *N* = 20• 15 females, 5 males• Age range: NR• Mean age: 31.95: • Work experience range: 1–22 years• Mean work experience: 7.25	Semi-structured in-depth telephone interviews.	Conventional content analysis	• Death anxiety, anxiety due to the disease, anxiety caused by corpse burial, fear of infecting the family, fear of being contaminated.• Problem related to the personal protective equipment, conflict between fear and conscience.
(Gao et al., 2020)ChinaHospitals	To explore nurses’ experiences regarding shift patterns while providing front-line care for COVID −19 patients in isolation wards of hospitals in Shanghai and Wuhan during the novel coronavirus pandemic.	•Qualitative exploratory descriptive design•Phenomen-ological research approach	• Purposive sampling• Nurses: *N* = 14• 13 females, 1 male• Age range: 24–43• Mean age: 33.5• Work experience range: 2–23• Mean work experience: 11.8	Semi-structured, in-depth interviews	Colaizzi's method	• Assess the competency of nurses to assign nursing work scientifically and reasonably.• reorganise nursing workflow to optimize shift patterns.• Communicate between managers and front -line nurses to humanize shift patterns.• Nurses’ various feelings and views on shift patterns.
(Góes et al., 2020)BrazilHospitals	To identify the challenges faced by pediatric nursing workers in the face of the COVID-19 pandemic.	Qualitative study	• Snowball technique• Nurses: *N* = 26• Age range: NR• Mean age: 33.1• Mean work experience: 12.3 • Work experience range: NR	Semi-structured electronic form of surveys	Lexicographic analysis	• Different challenges concerning the COVID-19 pandemic from the perspective of pediatric nursing workers.• A lack of protective equipment, training, diagnostic tests, and knowledge/information concerning the disease.• A lack of nursing workers and a lack of appreciation for the profession.
(Hou et al., 2020)ChinaHospitals	To explore the preparedness of the emergency department in a tertiary hospital in Taiyuan, Shanxi province, from the nurses’ perspectives during the COVID-19 outbreak.	• Qualitative Study• Husserl descriptive phenomen-ological approach	• Purposive sampling• Nurses: *N* = 12• 9 females, 3 males• Age range: 18–40• Mean age: 30.42• Work experience range: <1- >10• Mean work experience: NR	Semi-structured interviews	Colaizzi 7-step data analysis method	• Organizational preparedness.• Personal preparedness.• Patient and family preparedness. • Deficiencies and challenges.
(Jia et al., 2021)ChinaCOVID-19 designated units	To examine the ethical challenges encountered by nurses caring for patients with the novel coronavirus pneumonia (COVID-19) and share their coping styles to ethical conflicts and dilemmas, and to provide nurses with suggestions and support regarding promotion of their mental health.	Descriptive qualitative study	• Subsequent sampling• Nurses: *N* = 18• 13 females, 5 males• Age range: 24–43• Mean age: NR• Work experience range: 3–22• Mean work experience: NR	Structured in-depth interviews	Content analysis method	• The major ethical challenges encountered by nurses came from patients, inequality, professional ethics, and job competency. • The coping styles included active control and planning, seeking support.• The ethical challenges in nursing COVID-19 patients and their positive coping styles have impacts on the nurses’ career of specialised nursing skills, scientific research ability, and management skills.
(Kackin et al., 2020)TurkeyCOVID-19 wards	To determine the experiences and psychosocial problems among nurses caring for COVID-19 patients in Turkey	• Qualitative Study• Descriptive phenomen-ology	• Purposive sampling method• Nurses: *N* = 10• 8 females, 2 males• Age range: 24–40• Mean age: NR• Work experience range: NR• Mean work experience: NR	Individual, in-depth interview	Colaizzi's phenomenological analysis	• The nurses caring for COVID-19 patients in Turkey were negatively affected in psychological and social terms by the pandemic. • Short-term coping strategies and required psychosocial support and resource management. • The quality of patient care was negatively affected, and ethical dilemmas emerged. • The nurses felt fear and anxiety, and they showed depressive symptoms. • Nurses used short-term coping strategies to combat the negative effects of the COVID-19 pandemic, and needed psychosocial support and resource management.
(Kalateh et al., 2020)IranHospitals	To investigate the perceptions and experiences of nurses in the face of coronavirus outbreaks.	Qualitative study	• Purposive sampling technique• Nurses: *N* = 24• Age range: NR• Mean age: NR• Work experience range: NR• Mean work experience: NR	Semi-structured interviews	Inductive and deductive thematic analysis	• No clear understanding of the new virus. • Unpreparedness, the perceived risk, family protection, and social stigma. • Professional commitment. • Urgent preparedness of facilities in such outbreaks is inevitable. • Psychosocial support of nurses and their families and strengthening their sacrificial commitments are proposed in these conditions.
(Q Liu et al., 2020)ChinaHospitals	To describe the experiences of these physicians and nurses caring for COVID-19 in the early stages of the outbreak.	• Qualitative Study• Empirical phenomen-ological approach	• Purposive and snowball sampling• Nurses: *N* = 9• 7 females, 2 males• Age range: 22–36• Mean age: NR• Work experience range: 2–13• Mean work experience: NR	Semi-structured, in-depth telephone interviews	Haase's adaptation of Colaizzi's method	• Nurses had a crucial role in providing intensive care and assisting with activities of daily living. • Working in a new context, exhaustion due to heavy workloads and protective gear.• The fear of becoming infected and infecting others.• The feeling of powerlessness to handle patients’ conditions, and managing relationships. • Sources of social support and self-management strategies to cope with the situation.
(YE Liu et al., 2020)ChinaHospitals	To explore the experiences of front-line nurses combating the coronavirus disease-2019 epidemic.Research question: “What were the experiences of front-line nurses combating COVID-19?”	Qualitative Study	• Purposive sampling method• Nurses: *N* = 15• 10 females, 5 males• Age range: NR• Mean age: 27.83• Work experience range: NR• Mean work experience: 7.30	Semi-structured in-depth individual interviews	Content analysis methods	• Psychological and physical difficulties that nurses experienced. • Nurses played a crucial role during the pandemic.
(Sheng et al., 2020)ChinaHospitals	To explore the influence of experiences of involvement in the COVID-19 rescue task on professional identity among Chinese nurses from a qualitative method perspective.	• Qualitative part of a sequential mixed-method study• Empirical phenomen-ological approach	• Purposeful sampling approach• Nurses: *N* = 14• 11 females, 3 males• Age range: 23–40• Mean age: 32• Work experience range: 1–23• Mean work experience: NR	Semi-structured, audio-recorded, face-to-face interviews	Colaizzi's method of phenomen-ological analysis	• The main factors affecting the professional identity of rescue nurses. • The experiences of involvement in epidemic rescue tasks were described as facing complex challenges. • The negative impact on nurses' professional identity. • Nurses got unexpected professional benefits from the special experiences and improved their professional identity.
(Sun et al., 2020)ChinaHospitals	To understand the psychological experience of nurses participating in nursing COVID-19 patients.	• Qualitative Study• Colaizzi's phenomen-ological method	• Purposeful sampling method• Nurses: *N* = 20• 17 females, 3 males• Age range: 25–49• Mean age: 30.60• Work experience range: 1–28• Mean work experience: 5.85	Semi-structured interviews	Colaizzi's phenomen-ological analysis method	• Negative emotions present in the early stage consisting of fatigue. • Discomfort, and helplessness were caused by high-intensity work, fear and anxiety, and concern for patients and family members. • Self-coping styles. • The growth under pressure.
(Tan et al., 2020)ChinaHospitals	To describe, interpret, and understand the real feelings of first-line clinical nurses, their needs during clinical first-line work, and the problems they face, and to develop recommendations for solutions to these problems.	• Qualitative Study• Phenomen-ological method	• Purposive sampling• Nurses: *N* = 30• 24 females, 6 males• Age range: 24–47• Mean age: 31.23• Work experience range: 2–25• Mean work experience: 9.10	Semi-structured interviews by face-to-face, by telephone, and by WeChat over voice or video	Content analysis	• The difficulties related to labor shortages and a lack of protective equipment and experience. • The needs of clinical first-line nurses identified.
(Zhang et al., 2020)ChinaHospitals	To examine the psychological experience and change process of nurses in the epicenter of COVID-19 and to provide strategies that nurses could use to handle their stress.	Qualitative descriptive study	• Purposive sampling method• Nurses: *N* = 23• 18 females, 5 males• Age range: 23–40• Mean age: 31.5• Work experience range: 2–20• Mean work experience: 7.58	Semi-structured interviews	Colaizzi's method of data analysis	• The psychological change process of frontline nurses had three stages, early, middle, and later stages. • The psychological characteristics were ambivalence, emotional exhaustion, and energy renewal, respectively. • Nurse leaders were engaged in facilitating frontline nurses’ psychological adaptation.
(Deliktas et al., 2021)TurkeyHospital	To explore Turkish nurses’ experiences of working at COVID-19 pandemic units.	• Qualitative Study• Classic grounded theory methodology	• Purposive sampling and theoretical sampling• Nurses: *N* = 15• 14 females, 1 male• Age range: 21–39• Mean age: NR• Work experience range: 7 months-22 years• Mean work experience: NR	In-depth telephonic interviews	Constant comparative method	• Difficulties with the unknown. • Felt strengthened to have a positive impact on patients’ lives. • Different emotional responses. • Resources to empower nurses to cope with the struggle.• Challenges during the coping process. • Affected nurses’ views on lives, psychological symptoms and social isolation.
(Muz and Erdoğan, 2021)TurkeyHospitals	To reveal the physical, psychological, social and professional experiences of nurses caring for patients with COVID-19 at pandemic wards and intensive care units in Turkey.	• Qualitative study• Heidegger's phenomen-ological hermeneutic scientific approach	• Purposive sampling method• Nurses: *N* = 19• 17 females, 2 males• Age range: 23–40• Mean age: NR• Work experience range: 1–18 years• Mean work experience: NR	Semi-structured interview	Colaizzi's seven-step data analysis method	• The fear of contamination and contagion. • Changing working conditions and routines during the pandemic, and constant information updates about the virus.• Nurses felt unprepared for the pandemic.
(Coşkun and Günay, 2021)TurkeyHospitals	To examine the experiences and feelings of parent nurses who care for COVID-19 patients. Research questions: “What are the feelings of nurses who are working and must be away from their children in this pandemic?” and “What are the experiences of nurses working during the COVID-19 pandemic?”	Qualitative descriptive study	• Purposeful sampling method• Nurses: *N* = 26• 16 females, 10 males• Age range: 29–37• Mean age: NR• Work experience range: 1–12• Mean work experience: NR	Surveys with open-ended questions	Content analysis method	• The fear of transmitting the infection to their own children. • Nurses felt guilty for being away from their children.• Nurses worried about their children.
(N Galehdar et al., 2020)IranHospitals	To explore nurses' perceptions towards taking care of patients with this disease.	Qualitative study	• Purposeful sampling method• Nurses: *N* = 13• 11 females, 2 males• Age range: NR• Mean age: 33• Mean work experience: 13• Work experience range: NR	Semi-structured in-depth telephone interviews	Conventional content analysis approach	• Challenges during taking care of patients with COVID-19. • Decreased quality of care. • Improved nurses' occupational status and morale and deepened the understanding of the nursing profession.
(Sethi et al., 2020)PakistanHospitals	To explore the impact of Coronavirus disease pandemic on nurses and the associated challenges.	Descriptive cross-sectional survey	• Snowball sampling• Nurses: *N* = 210• 116 females, 94 males• Age range: 21–50• Mean age: NR• Work experience range: NR• Mean work experience: NR	Questionnaire with open-ended questions	Qualitative content analysis	• Anxiety, distress, and depression. • The exceptional workload. • Improved self-esteem and self-image in the society.• Some family, academia, clinical services, and public related challenges were identified.
(Fernández-Castillo et al., 2021)SpainHospital	To explore and document the experiences of nurses working in an intensive care unit where patients diagnosed with SARS-CoV2 infection were treated during COVID-19 pandemic.	Descriptive qualitative study	• Homogeneous purposive sampling• Nurses: *N* = 17• 11 females, 6 males• Age range: 31–54• Mean age: NR• Work experience range: 2–25• Mean work experience: NR	Semi-structured interviews	Inductive content analysis	• The provision of care has been influenced by the isolation of patients and the fear experienced by professionals. • Dehumanization of care. • Physical consequences, emotional and mental hardship.• Staff were recruited to units during the pandemic.• Nurses generated greater anxiety and concern to cope with the workload. • Good support related to work.• The help from teams.
(Ohta et al., 2020)JapanCOVID-19 ward in hospital	To examine nurses' changing perceptions of preparing for COVID-19 and working in COVID-19 wards.	Qualitative study	• Nurses: *N* = 16• Age range: NR• Mean age: NR• Work experience range: NR• Mean work experience: NR	Ethnography and semi-structured interviews	Grounded theory	• Nurses working in COVID-19 wards had previously felt unpredictable fear regarding COVID-19. • Nurses established and improved methods for approaching COVID-19, acquired confidence at work, and regained sympathy for patients. • Working in the COVID-19 ward negatively affected nurses’ activities outside of the ward.
(Lee and Lee, 2020)South KoreaHospital	To explore the experiences of COVID-19-designated hospital nurses in South Korea who provided care for patients based on their lived experiences.	• Qualitative study• Giorgi's phenomeno-logical method	• Purposive and snowball sampling• Nurses: *N* = 18• 18 females, 0 males• Age range: 20–49• Mean age: NR• Work experience range: 2–22 years• Mean work experience: 7.44	In-depth interviews	Giorgi descriptive phenomenology method	• Nurses in COVID-19 hospital were pushed into the forefront of the pandemic.• Lack of preparation. • Nurses experienced changes at work and home due to COVID-19.• Nurses’ motivation decreased as their efforts were not properly recognized. • Exhaustion for the protracted pandemic.• Negative emotions. • Social support from peers, family, friends, patients, and the public. • The positive meaning from work and self-growing.
(Goh et al., 2020)SingaporeHospitals	To explore the experiences of registered nurses working in tertiary hospitals during the COVID-19 pandemic.	Qualitative study	• Purposive sampling and snowball sampling• Nurses: *N* = 17 • 11 females, 6 males• Age range: 22–67• Mean age: 32.6• Work experience range: 2–19• Mean work experience: NR	Semi-guided open-ended interviews	Thematic analysis	• Physical and psychological challenges relating to working conditions of the hospital.• The professional role of nurses.• The support for nurses from their family, friends and leaders to persevere and overcome the challenges of COVID-19.• The nurses demonstrated resilience and professionalism. • The importance of a robust support system.
(Bennett et al., 2020)UKHospitals	To gain insight into the experiences and concerns of front-line National Health Service (NHS) workers while caring for patients with COVID-19.	Qualitative study	• Snowball sampling on Twitter• Nurses: *N* = 13• Age range: NR• Mean age: NR• Work experience range: NR• Mean work experience: NR	Digital audio recording	Inductive thematic analysis	• The aspects of being the experience and psychological consequence of trauma. • The positive experiences.• The significant emotional toll. • Strained relationships between frontline staff, their families, management and government.
(Vindrola-Padros et al., 2020)UKHospitals	To explore the perceptions and experiences of HCWs in relation to COVID-19 and care delivery models implemented to deal with the pandemic in the UK.	Rapid appraisal method	• Purposive sampling• Nurses: *N* = 3• Age range: NR• Mean age: NR• Work experience range: NR• Mean work experience: NR	In-depth, semi-structured telephone interviews	Framework analysis	• Limited PPE and lack of routine testing created anxiety and distress. • Incorrect size and overheating of PPE complicated routine work. • Lack of training. • Positive aspects included solidarity between colleagues, the establishment of well-being support structures and feeling valued by society.
(Okediran et al., 2020)NigeriaIsolation centers	To explore and describe the experiences of health-care workers (HCWs) who were involved in the COVID-19 response at the beginning of the COVID-19 outbreak in Nigeria.	Qualitative study	• Purposive and snowballing techniques• Nurses: *N* = 4• 4 females, 0 males• Age range: 29–51• Mean age: NR• Work experience range: 6–30• Mean work experience: NR	Face-to-face in-depth interviews	Colaizzi's phenomenological method	• The optimism regarding COVID-19 care. • Difficulties working in a new environment. • Limited resources. • Coping through the available support systems. • The feelings varied from pleasure on patients’ recovery to distress following patients’ demise. • The need for increased psychosocial support, and adequate provision of material and financial support.

*N = Number of participants; NR = Not reported.

### 2.6 Data synthesis

Initially, a descriptive summary analysis was supported by Table 4 and presented as ‘Characteristics of the studies’ in the result section. Considering that all studies used a qualitative analytical approach and most of them presented thematic findings, thematic synthesis, inspired by Thomas and Harden, was an appropriate approach to deliver key messages from primary data and to generate higher-level themes (Nicholson et al., 2016). First, the articles were read and re-read to develop a sense of the studies as a whole. The synthesis process was an inductive three-stages approach that began with collecting findings of each primary study and freely coding the texts line-by-line according to their meaning and content (Thomas and Harden, 2008). Following this stage came the development of descriptive themes which involved translating concepts from one study to another by combining codes, and then creating a hierarchical structure by considering similarities and differences between codes (Thomas and Harden, 2008). The final stage consisted of generating analytical themes from the content of the primary studies and determining key messages through descriptive themes according to the review aim (Thomas and Harden, 2008). The thematic synthesis led to the identification of three themes that describe the psychosocial experiences of frontline nurses working in hospital-based settings during the COVID-19 pandemic: ‘Nurses' emotional, mental and physical reactions to COVID-19’, ‘Internally and externally supported coping strategies’, and ‘A call for future help and support’.

## 3 Results

### 3.1 Characteristics of the studies

Twenty-eight study characteristics were included in Table 4. The studies were conducted in China (Fan et al., 2020, Gao et al., 2020, Hou et al., 2020, Jia et al., 2021, Q Liu et al., 2020, YE Liu et al., 2020, Sheng et al., 2020, Sun et al., 2020, Tan et al., 2020, Zhang et al., 2020), Iran (Eftekhar et al., 2020, N Galehdar et al., 2020, Kalateh et al., 2020, N Galehdar et al., 2020), Turkey (Kackin et al., 2020, Deliktas et al., 2021, Muz and Erdoğan, 2021, Coşkun and Günay, 2021), UK (Bennett et al., 2020, Vindrola-Padros et al., 2020), USA (Arnetz et al., 2020), Brazil (Góes et al., 2020), Pakistan (Sethi et al., 2020), Spain (Fernández-Castillo et al., 2021), Japan (Ohta et al., 2020), South Korea (Lee and Lee, 2020), Singapore (Goh et al., 2020), and Nigeria (Okediran et al., 2020). The setting for 25 of the included studies were hospitals, two were in COVID-19 designated wards, and one study was in a COVID-19 isolation center. There were a total of 1141 nurses included in the review data. The reported ages of nurses ranged from 18 to 65 years or older, and the reported work experience ranged from 7 months to 30 years.

Twenty-five studies used a qualitative study design. Two studies used a cross-sectional design and one study used a rapid appraisal method, all of which used a qualitative approach to collect, analyze, and report data with appropriate rigor. Theoretical methodologies adopted by the studies were phenomenology (Gao et al., 2020, Hou et al., 2020, Kackin et al., 2020, Q Liu et al., 2020, Sheng et al., 2020, Sun et al., 2020, Tan et al., 2020, Muz and Erdoğan, 2021, Lee and Lee, 2020), and grounded theory (Deliktas et al., 2021). Data collection methods included interviews (Eftekhar et al., 2020, Fan et al., 2020, N Galehdar et al., 2020, Gao et al., 2020, Hou et al., 2020, Jia et al., 2021, Kackin et al., 2020, Kalateh et al., 2020, Q Liu et al., 2020, YE Liu et al., 2020, Sheng et al., 2020, Sun et al., 2020, Tan et al., 2020, Zhang et al., 2020, Deliktas et al., 2021, Muz and Erdoğan, 2021, N Galehdar et al., 2020, Fernández-Castillo et al., 2021, Ohta et al., 2020, Lee and Lee, 2020, Goh et al., 2020, Vindrola-Padros et al., 2020, Okediran et al., 2020), surveys or questionnaires (Arnetz et al., 2020, Góes et al., 2020, Coşkun and Günay, 2021, Sethi et al., 2020), and digital audio recording (Bennett et al., 2020). Seven of 23 study interviews were conducted via voice or video (Eftekhar et al., 2020, N Galehdar et al., 2020, Q Liu et al., 2020, Tan et al., 2020, Deliktas et al., 2021, N Galehdar et al., 2020, Vindrola-Padros et al., 2020), and the others were face-to-face interviews (Fan et al., 2020, Gao et al., 2020, Hou et al., 2020- (Kalateh et al., 2020, YE Liu et al., 2020, Sheng et al., 2020, Sun et al., 2020, Zhang et al., 2020, Muz and Erdoğan, 2021, Fernández-Castillo et al., 2021, Ohta et al., 2020, Lee and Lee, 2020, Goh et al., 2020, Okediran et al., 2020). Nine different analytical methods were utilised: content analysis (Arnetz et al., 2020, N Galehdar et al., 2020, Jia et al., 2021, YE Liu et al., 2020, Tan et al., 2020, Coşkun and Günay, 2021, N Galehdar et al., 2020, Sethi et al., 2020, Fernández-Castillo et al., 2021), thematic analysis (Eftekhar et al., 2020, Fan et al., 2020, Kalateh et al., 2020, Goh et al., 2020, Bennett et al., 2020), descriptive phenomenological method (Gao et al., 2020, Hou et al., 2020, Kackin et al., 2020, Q Liu et al., 2020, Sheng et al., 2020, Sun et al., 2020, Zhang et al., 2020, Muz and Erdoğan, 2021, Okediran et al., 2020), lexicographic analysis (Góes et al., 2020), constant comparative method (Deliktas et al., 2021), grounded theory (Ohta et al., 2020), descriptive phenomenology method (Lee and Lee, 2020), and framework analysis (Vindrola-Padros et al., 2020).

### 3.2 Quality assessment of included studies

Based on the outcomes of CASP, eight studies met the requirements of ten questions (Arnetz et al., 2020, Eftekhar et al., 2020, Hou et al., 2020, Kackin et al., 2020, Tan et al., 2020, Deliktas et al., 2021, Bennett et al., 2020, Vindrola-Padros et al., 2020), and 20 studies met the requirements of nine questions. Two studies did not clearly elaborate their recruitment strategies (N Galehdar et al., 2020, Ohta et al., 2020), 16 studies did not critically examine the researchers’ roles or adequately consider the relationships between researchers and participants in their formulation of research questions or data collection (Fan et al., 2020, Gao et al., 2020, Góes et al., 2020, Jia et al., 2021, Kalateh et al., 2020, Q Liu et al., 2020, YE Liu et al., 2020, Sheng et al., 2020, Zhang et al., 2020, Muz and Erdoğan, 2021, Coşkun and Günay, 2021, N Galehdar et al., 2020, Fernández-Castillo et al., 2021, Lee and Lee, 2020, Goh et al., 2020, Okediran et al., 2020), and two studies did not provide in-depth descriptions or discussions of data analysis (Sun et al., 2020, Sethi et al., 2020). All selected studies were deemed to have appropriate methodological rigor (Table 3).

### 3.3 Nurses' emotional, mental and physical reactions to COVID-19

The studies described various emotional states caused by the COVID-19 pandemic (Arnetz et al., 2020, Eftekhar et al., 2020, N Galehdar et al., 2020, Gao et al., 2020, Góes et al., 2020, Kackin et al., 2020- (Bennett et al., 2020). The most common emotional state was fear, especially at the onset of the COVID-19 pandemic, although anxiety and stress also appeared to affect the nurses’ emotions. The fear nurses reported was mainly a fear of being infected in their frontline work (Arnetz et al., 2020, Eftekhar et al., 2020, N Galehdar et al., 2020, Gao et al., 2020, Góes et al., 2020, Kackin et al., 2020, Kalateh et al., 2020, Q Liu et al., 2020, YE Liu et al., 2020, Sheng et al., 2020, Sun et al., 2020, Tan et al., 2020, Deliktas et al., 2021, Coşkun and Günay, 2021, N Galehdar et al., 2020, Goh et al., 2020, Bennett et al., 2020). During the outbreak, nurses who had close contact with patients experienced initial fears of being contaminated at work, and of becoming sick and dying (Arnetz et al., 2020, Eftekhar et al., 2020, N Galehdar et al., 2020, Gao et al., 2020, Góes et al., 2020, Q Liu et al., 2020, YE Liu et al., 2020, Sheng et al., 2020, Tan et al., 2020, Deliktas et al., 2021, N Galehdar et al., 2020, Goh et al., 2020, Bennett et al., 2020). Some nurses reported becoming overly vigilant in terms of detecting minor symptoms related to COVID-19 and developed obsessive thoughts of being infected (Coşkun and Günay, 2021, Lee and Lee, 2020). Some nurses reported that the infection or death of other medical staff exacerbated their fear, creating serious anxiety and stress (Kalateh et al., 2020, Sheng et al., 2020, Sun et al., 2020).

In addition to the fear of oneself being infected, nurses also expressed concerns about transmitting the infection (Arnetz et al., 2020, Eftekhar et al., 2020, N Galehdar et al., 2020, Góes et al., 2020, Kackin et al., 2020, Kalateh et al., 2020, Q Liu et al., 2020, YE Liu et al., 2020, Sheng et al., 2020, Sun et al., 2020, Tan et al., 2020, Coşkun and Günay, 2021, N Galehdar et al., 2020, Sethi et al., 2020, Fernández-Castillo et al., 2021, Goh et al., 2020, Bennett et al., 2020). This potential risk increased the nurses’ anxiety as they worried about being carriers of the virus and infecting their family members and loved ones. The nurses expressed a sense of guilt or self-blame for the infection or death of family members (Eftekhar et al., 2020, N Galehdar et al., 2020, N Galehdar et al., 2020). Nonetheless, the concerns regarding the nurses’ safety and lack of familiarity with and understanding of the nurses’ frontline work from family members increased the nurses’ psychological stress (Fan et al., 2020, Kackin et al., 2020, Sun et al., 2020), and some nurses chose to hide the truth about working frontline from their family members (Kackin et al., 2020).

In the face of the COVID-19 surge, studies reported that nurses lingered with a sense of unknown and uncertainty (Arnetz et al., 2020, Eftekhar et al., 2020, N Galehdar et al., 2020, Kackin et al., 2020, Kalateh et al., 2020, YE Liu et al., 2020, Zhang et al., 2020, Deliktas et al., 2021, Muz and Erdoğan, 2021, Fernández-Castillo et al., 2021, Ohta et al., 2020, Lee and Lee, 2020, Bennett et al., 2020). Early in the pandemic, since the disease was unprecedented and thus hardly known by the public and scientific authorities, nurses were working under stress resulting from the lack of scientific information available (N Galehdar et al., 2020, Góes et al., 2020). This ambiguous and unpredictable situation brought an unavoidable fear to frontline nurses (YE Liu et al., 2020, Tan et al., 2020). Information that differs from the nurses’ understanding of COVID19 and over-information from the media were reported as some of the most stressful factors affecting the nurses’ emotions (Arnetz et al., 2020, Eftekhar et al., 2020, N Galehdar et al., 2020, Góes et al., 2020, Fernández-Castillo et al., 2021, Ohta et al., 2020, Lee and Lee, 2020). They felt unable to disconnect from this awkward situation and were extremely anxious about the varied and uncertain content being spread (Fernández-Castillo et al., 2021, Ohta et al., 2020). Additionally, the nurses reported concerns about the future in relation to work and personal life as the pandemic continued (Lee and Lee, 2020), including concerns about neglecting patients with other diseases (Eftekhar et al., 2020), the ability to deal with COVID-19 patients (Ohta et al., 2020), the control of the current pandemic (YE Liu et al., 2020, Deliktas et al., 2021), and future financial situation of nurses and their families (Eftekhar et al., 2020).

Due to the sudden nature of the outbreak, the number of patients in hospitals increased sharply, while nurses, particularly those with special knowledge of intensive care and respiratory diseases, were understaffed to handle such heavy workloads (Góes et al., 2020, Kackin et al., 2020, Q Liu et al., 2020, Tan et al., 2020, Sethi et al., 2020, Goh et al., 2020). The shortage of workforce led to excessive workloads and longer work shifts for nurses to make up for the shortfalls (Eftekhar et al., 2020, Fan et al., 2020, Gao et al., 2020, Q Liu et al., 2020, Sheng et al., 2020, Tan et al., 2020, Deliktas et al., 2021, N Galehdar et al., 2020, Sethi et al., 2020, Fernández-Castillo et al., 2021, Lee and Lee, 2020, Goh et al., 2020). Nurses reported concerns about the quality of care provided as they experienced physical fatigue and psychological stress (Eftekhar et al., 2020, Fan et al., 2020, Gao et al., 2020, Q Liu et al., 2020, Sheng et al., 2020, Tan et al., 2020, N Galehdar et al., 2020). Furthermore, nurses expressed feelings of unfairness regarding the occupational division of labor (Jia et al., 2021, Kackin et al., 2020, Sheng et al., 2020, Deliktas et al., 2021, Lee and Lee, 2020). They were dissatisfied with the unequal exposure to infectious environments compared to other medical staff (Jia et al., 2021, Kackin et al., 2020, Sheng et al., 2020, Lee and Lee, 2020), and they felt demoralised by being excluded from decisions about the treatment or care of COVID-19 patients (Deliktas et al., 2021).

Unfamiliar workflows and environments generated great physical and mental challenges for the nurses (Fan et al., 2020, N Galehdar et al., 2020, Gao et al., 2020, Q Liu et al., 2020, Sun et al., 2020, Tan et al., 2020, Zhang et al., 2020, Coşkun and Günay, 2021, Sethi et al., 2020, Lee and Lee, 2020, Okediran et al., 2020). The nurses described that they had to take care of critically ill patients and handle complex conditions while the operating procedures were still unclear (Gao et al., 2020, Sun et al., 2020, Tan et al., 2020, Zhang et al., 2020, Coşkun and Günay, 2021, Lee and Lee, 2020). Studies reported that nurses working in these intense situations experienced a range of negative mental and emotional reactions, including stress (Fan et al., 2020, N Galehdar et al., 2020, Gao et al., 2020), anxiety (N Galehdar et al., 2020, Sun et al., 2020, Zhang et al., 2020, Okediran et al., 2020), sense of oppression (Q Liu et al., 2020), feelings of suffocation and depression (Bennett et al., 2020). Nurses described isolation wards as an oppressive and stressful place, perceived as full of contamination risks, which led to emotional distress (Fan et al., 2020, N Galehdar et al., 2020, Q Liu et al., 2020, Sethi et al., 2020). The nurses were psychologically unprepared to work on the frontline of a pandemic (N Galehdar et al., 2020, Góes et al., 2020, Muz and Erdoğan, 2021, Ohta et al., 2020, Lee and Lee, 2020, Bennett et al., 2020), and the healthcare system's lack of preparedness was manifested through incomplete, unclear, and continuously-evolving guidelines (Eftekhar et al., 2020, N Galehdar et al., 2020, Kackin et al., 2020, Deliktas et al., 2021, Sethi et al., 2020, Lee and Lee, 2020, Goh et al., 2020, Bennett et al., 2020, Vindrola-Padros et al., 2020). Nurses reported that they were confused and dissatisfied about the changing guidelines because it made their work inconsistent and lacking in adherence (Eftekhar et al., 2020, Kackin et al., 2020, Sethi et al., 2020, Vindrola-Padros et al., 2020), which caused anxiety and psychological fatigue (N Galehdar et al., 2020, Deliktas et al., 2021, Lee and Lee, 2020, Goh et al., 2020, Bennett et al., 2020). Moreover, the insufficient knowledge not only hindered nurses’ performance in specialised care (N Galehdar et al., 2020, Góes et al., 2020, Jia et al., 2021, Q Liu et al., 2020, Tan et al., 2020, N Galehdar et al., 2020, Sethi et al., 2020, Fernández-Castillo et al., 2021), but it also caused anxiety about the effectiveness of treatment as well as self-protection (YE Liu et al., 2020, Sheng et al., 2020, Sun et al., 2020, Deliktas et al., 2021, Coşkun and Günay, 2021).

The usage of PPE came with physical, psychological, and professional challenges (Eftekhar et al., 2020, N Galehdar et al., 2020, Gao et al., 2020, Góes et al., 2020, Kackin et al., 2020, Q Liu et al., 2020, YE Liu et al., 2020, Sheng et al., 2020, Sun et al., 2020, Tan et al., 2020, Zhang et al., 2020, Muz and Erdoğan, 2021, N Galehdar et al., 2020, Fernández-Castillo et al., 2021, Lee and Lee, 2020, Goh et al., 2020, Okediran et al., 2020). Nurses experienced physical discomfort (Eftekhar et al., 2020, Gao et al., 2020, Kackin et al., 2020, Q Liu et al., 2020, YE Liu et al., 2020, Sun et al., 2020, Tan et al., 2020, Zhang et al., 2020, Muz and Erdoğan, 2021, N Galehdar et al., 2020, Okediran et al., 2020), and restrictions in terms of avoidance of eating and drinking while wearing PPE (N Galehdar et al., 2020, Gao et al., 2020, Q Liu et al., 2020, Sun et al., 2020). Altogether, those uncomfortable feelings caused physical exhaustion and psychological stress in frontline nurses (Eftekhar et al., 2020, N Galehdar et al., 2020, Gao et al., 2020, Góes et al., 2020, Kackin et al., 2020, YE Liu et al., 2020, Sheng et al., 2020, Fernández-Castillo et al., 2021, Goh et al., 2020). After adopting protective measures for a period of time, the nurses became less vigilant due to physical and mental exhaustion (Eftekhar et al., 2020, N Galehdar et al., 2020, Jia et al., 2021). Moreover, nurses reported that PPE was an obstacle to the efficiency of nursing procedures because it hindered communication, visibility, and movement (Q Liu et al., 2020, YE Liu et al., 2020, Sun et al., 2020, Lee and Lee, 2020, Goh et al., 2020). PPE concealed the nurses’ identity in their contact with patients, which impaired nurses’ self-esteem and led to a sense of alienation (N Galehdar et al., 2020). Besides, the shortage of protective materials exacerbated the nurses’ anxiety and affected their care performance (Arnetz et al., 2020, Eftekhar et al., 2020, Góes et al., 2020, Kalateh et al., 2020, Q Liu et al., 2020, Tan et al., 2020, N Galehdar et al., 2020, Sethi et al., 2020, Fernández-Castillo et al., 2021, Okediran et al., 2020).

The nurses expressed strong psychological and emotional stress associated with witnessing the suffering and death of patients (Arnetz et al., 2020, Eftekhar et al., 2020, Fan et al., 2020, N Galehdar et al., 2020, Kackin et al., 2020, Q Liu et al., 2020, YE Liu et al., 2020, Sheng et al., 2020, Tan et al., 2020, Zhang et al., 2020, N Galehdar et al., 2020, Okediran et al., 2020). The handling of the deceased with COVID-19 was also difficult for nurses to accept psychologically (Kackin et al., 2020, Q Liu et al., 2020). Scenes witnessed by frontline nurses caused a range of psychological and mental distress including frustration (Arnetz et al., 2020, Eftekhar et al., 2020), depression (Q Liu et al., 2020, Tan et al., 2020), anxiety (Tan et al., 2020), fear (N Galehdar et al., 2020, Kackin et al., 2020), stress (N Galehdar et al., 2020, YE Liu et al., 2020, N Galehdar et al., 2020), sense of helplessness (Eftekhar et al., 2020), and other symptoms of PTSD (Eftekhar et al., 2020). Furthermore, the nurses’ mental and emotional states fluctuated according to the patients’ condition (Q Liu et al., 2020, Zhang et al., 2020, Muz and Erdoğan, 2021, N Galehdar et al., 2020, Okediran et al., 2020).

Despite the fact that nurses did their best to treat patients, there was a relatively high mortality rate and lower instances of improvement of patients’ condition (Eftekhar et al., 2020, Q Liu et al., 2020, Sheng et al., 2020, Fernández-Castillo et al., 2021). Nurses felt that they were unable to provide patients with adequate support (Arnetz et al., 2020, Eftekhar et al., 2020, Fan et al., 2020, N Galehdar et al., 2020, Jia et al., 2021, Sheng et al., 2020, Sun et al., 2020, Muz and Erdoğan, 2021, Fernández-Castillo et al., 2021). Accordingly, nurses, particularly those working in intensive care units, expressed a sense of helplessness and powerlessness (Arnetz et al., 2020, Eftekhar et al., 2020, Fan et al., 2020, N Galehdar et al., 2020, Q Liu et al., 2020, Sheng et al., 2020, Tan et al., 2020, Fernández-Castillo et al., 2021), as well as a sense of losing control over the patients’ condition (Eftekhar et al., 2020). Nurses acknowledged an insufficiency in the psychological and emotional care they delivered to patients and their family members due to the lack of relevant knowledge and an inner fear of contagion (Jia et al., 2021, Sheng et al., 2020, Deliktas et al., 2021, Muz and Erdoğan, 2021, N Galehdar et al., 2020). In addition, nurses retained hesitation in coming close to patients due to the fear of contamination (Jia et al., 2021, Kackin et al., 2020, Muz and Erdoğan, 2021), and they reduced the frequency and speed of nursing activities to protect their own safety (Jia et al., 2021). This perceived inadequacy of care triggered moral distress in nurses, and nurses were under pressure due to the decline in quality of care (Kackin et al., 2020, Deliktas et al., 2021, Muz and Erdoğan, 2021, Coşkun and Günay, 2021, Fernández-Castillo et al., 2021). Besides, ethical dilemmas in care caused by COVID-19 also created frustration and moral distress for nurses (Hou et al., 2020, Jia et al., 2021, Fernández-Castillo et al., 2021). Patients’ rights, such as the right to information, seemed to be neglected, and humanization of care could not be maintained due to the restrictions of PPE and strict isolation procedures (Fernández-Castillo et al., 2021).

The stress accumulated during frontline work resulted in negative effects on nurses’ physical, mental, and psychological well-being. Since the nurses had gone through excessive occupational pressure and continuous states of emotional agitation under the prolonged pandemic (Jia et al., 2021, Zhang et al., 2020, Lee and Lee, 2020), many nurses manifested signs of mental breakdown such as crying (Jia et al., 2021), depression (Eftekhar et al., 2020, Lee and Lee, 2020), high-level of aggression (Zhang et al., 2020), and the emergence of trauma-related symptoms (Eftekhar et al., 2020, Bennett et al., 2020), such as sleep disorders (YE Liu et al., 2020, Zhang et al., 2020), recurrent scenes of patients dying (Eftekhar et al., 2020), and emotional numbing (Bennett et al., 2020). In addition, physical decline was reported among nurses, including symptoms of fatigue (Sun et al., 2020, Zhang et al., 2020, Lee and Lee, 2020), headaches and dizziness (Lee and Lee, 2020).

### 3.4 Internally and externally supported coping strategies

Nurses reported that, as a result of the pandemic-associated living and working conditions, their daily routines had changed significantly in terms of eating, sleeping, and outdoor activities (Eftekhar et al., 2020, N Galehdar et al., 2020, Sun et al., 2020, Deliktas et al., 2021, Sethi et al., 2020, Lee and Lee, 2020, Okediran et al., 2020). Nurses were actively seeking for knowledge (Hou et al., 2020, Jia et al., 2021), adjusting their attitudes towards the situation and being optimistic about the faced challenges (Sun et al., 2020), avoiding overthinking about the current pandemic and remarks on social media (Kackin et al., 2020, Sun et al., 2020), and developing interests to distract themselves (Kackin et al., 2020, Sun et al., 2020), many of them found their own method to relieve anxiety and to psychologically normalize the pandemic realities (Eftekhar et al., 2020, Sun et al., 2020). It was reported that the improvement of knowledge about the disease, nursing skills, and protective measurements had increased nurses’ security and self-confidence in frontline work (Hou et al., 2020, Jia et al., 2021, Deliktas et al., 2021, Okediran et al., 2020), and greatly diminished their stress (Deliktas et al., 2021). Some nurses experienced changes to their perspectives on life, appreciating and cherishing life more than before (Q Liu et al., 2020, Deliktas et al., 2021). However, some nurses also expressed a sense of powerlessness in resisting stress and regulating psychological states (Jia et al., 2021).

Nurses viewed communication with family members as an additional psychosocial support that could encourage, motivate, and comfort them (Q Liu et al., 2020, Sun et al., 2020, N Galehdar et al., 2020, Goh et al., 2020, Okediran et al., 2020). Considering the potential risk of being a carrier, nurses restricted their social activities, and many of them had even isolated themselves from family members and friends (Eftekhar et al., 2020, N Galehdar et al., 2020, Kalateh et al., 2020, Muz and Erdoğan, 2021, Coşkun and Günay, 2021, Lee and Lee, 2020). As family members and friends used to be an important support system that nurses relied on (Arnetz et al., 2020), these new social restrictions and self-isolation suddenly and dramatically decreased nurses’ social and emotional relationships (Arnetz et al., 2020, Eftekhar et al., 2020, Kackin et al., 2020, Muz and Erdoğan, 2021, Sethi et al., 2020, Lee and Lee, 2020), which resulted in depression and loneliness for nurses during this adaptation process (Arnetz et al., 2020, Eftekhar et al., 2020, Muz and Erdoğan, 2021, Sethi et al., 2020). Nurses with children expressed a sense of anxiety and guilt for being apart from their young children while they took care of patients at work, and not being able to explain the fact about the pandemic to their children exacerbated their worries and stress (N Galehdar et al., 2020, Coşkun and Günay, 2021, N Galehdar et al., 2020). Nurses expressed additional stress related to the management of family-related issues, such as financial concerns due to the unemployment of family members (Arnetz et al., 2020, Sethi et al., 2020). Moreover, the adaptation of changed social dynamics brought additional challenges and difficulties to the nurses (Hou et al., 2020, Deliktas et al., 2021, Muz and Erdoğan, 2021, Sethi et al., 2020, Ohta et al., 2020, Lee and Lee, 2020). Nurses who worked in COVID-19 wards reported experiences of being alienated from staff in the other departments and excluded by society, which felt disappointing and difficult to deal with (Deliktas et al., 2021, Muz and Erdoğan, 2021, Ohta et al., 2020, Lee and Lee, 2020). People avoided approaching nurses and viewed them as potential threats (Hou et al., 2020, Deliktas et al., 2021, Muz and Erdoğan, 2021, Ohta et al., 2020, Lee and Lee, 2020). On social media platforms, people criticised the professional competence of nurses who became infected at work, and the nurses were maliciously judged by people's comments based on unreasonable social standards (Arnetz et al., 2020, Sethi et al., 2020, Lee and Lee, 2020). These pressures created a substantial psychological burden for nurses to bear. Besides, nurses also worried about discrimination against family members because of their working conditions (Ohta et al., 2020). Therefore, it was difficult for nurses to physically and mentally adapt to a pandemic-related personal life and social conditions (Okediran et al., 2020).

Regarding occupational coping, nurses reported that it was difficult to adjust to the work environment and coordinate as multidisciplinary teams in the early stage of the pandemic (Q Liu et al., 2020, Deliktas et al., 2021, Bennett et al., 2020, Okediran et al., 2020). The praise and affirmation from the public (YE Liu et al., 2020, Coşkun and Günay, 2021, Lee and Lee, 2020, Goh et al., 2020), patients’ understanding and appreciation (Lee and Lee, 2020), professional solidarity (Q Liu et al., 2020, Sheng et al., 2020, Sun et al., 2020, Zhang et al., 2020, Deliktas et al., 2021, Muz and Erdoğan, 2021, Fernández-Castillo et al., 2021, Lee and Lee, 2020, Goh et al., 2020), organisational support (Hou et al., 2020, Jia et al., 2021, Q Liu et al., 2020, Sheng et al., 2020, Zhang et al., 2020, Goh et al., 2020), and emotional support from the family (Q Liu et al., 2020, Sun et al., 2020, N Galehdar et al., 2020, Goh et al., 2020, Okediran et al., 2020), contributed to an increased motivation and psychological strength for nurses. Logistical support, such as providing protective supplies (Jia et al., 2021, Q Liu et al., 2020, Sheng et al., 2020), accommodations (Q Liu et al., 2020, Sheng et al., 2020), financial rewards (Q Liu et al., 2020, Sheng et al., 2020, Goh et al., 2020), and the professional training and psychological intervention provided by hospitals led to a sense of satisfaction for nurses (Jia et al., 2021, Zhang et al., 2020, Goh et al., 2020, Okediran et al., 2020). Meanwhile, trust and understanding within teams cultivated over time so that multidisciplinary cooperation became significantly and efficiently strengthened (Gao et al., 2020, Hou et al., 2020, Ohta et al., 2020). Nurses highlighted that the mutually supportive relationship with colleagues provided them with encouragement and a sense of safety (Q Liu et al., 2020, Sun et al., 2020, Deliktas et al., 2021, Muz and Erdoğan, 2021, Lee and Lee, 2020, Goh et al., 2020, Okediran et al., 2020). The care and understanding from managers helped alleviate physical and psychological stress (Hou et al., 2020, Zhang et al., 2020, Okediran et al., 2020). Through familiarisation and workplace support, nurses gradually overcame their fears and stress (Sun et al., 2020, Zhang et al., 2020, Ohta et al., 2020), and adapted to the new work routines (Eftekhar et al., 2020, Hou et al., 2020, Okediran et al., 2020). Consequently, this adaptation contributed to increased confidence of nurses in managing patients and helped in maintaining their mental well-being (Ohta et al., 2020, Okediran et al., 2020).

Nurses reported that their overall competency in responding to an infectious disease outbreak or public health emergency was improved (Eftekhar et al., 2020, Fan et al., 2020, Jia et al., 2021, Q Liu et al., 2020, Sheng et al., 2020, Muz and Erdoğan, 2021, Lee and Lee, 2020, Goh et al., 2020, Bennett et al., 2020). Nurses were grateful for having received valuable experiences of working frontline for the pandemic because their critical thinking and decision-making skills were enhanced, as well as their ability to cope with challenging situations (Jia et al., 2021, Vindrola-Padros et al., 2020). Nurses underlined the significant role of professional values in frontline work (Eftekhar et al., 2020, Fan et al., 2020, Góes et al., 2020, Hou et al., 2020, Kalateh et al., 2020, Q Liu et al., 2020, YE Liu et al., 2020, Sun et al., 2020, Tan et al., 2020, Zhang et al., 2020, Deliktas et al., 2021, Muz and Erdoğan, 2021, Coşkun and Günay, 2021, N Galehdar et al., 2020, Ohta et al., 2020, Lee and Lee, 2020, Goh et al., 2020, Bennett et al., 2020, Okediran et al., 2020). Professional commitment was the basis of their motivation and enthusiasm, and professional values supported their dedication to working on the frontline of the pandemic without hesitation and in spite of the risks (Hou et al., 2020, Kackin et al., 2020, Kalateh et al., 2020, YE Liu et al., 2020, Sun et al., 2020, Tan et al., 2020, Zhang et al., 2020, Deliktas et al., 2021, Coşkun and Günay, 2021, N Galehdar et al., 2020, Bennett et al., 2020, Okediran et al., 2020). However, some nurses expressed fear of these unavoidable duties when they were called to the frontline (Lee and Lee, 2020). Nurses made great efforts to provide high-quality care (Góes et al., 2020, Q Liu et al., 2020, N Galehdar et al., 2020, Ohta et al., 2020), maintain the essence of human care (Fan et al., 2020, N Galehdar et al., 2020), and provide integral assistance to patients and family members (Góes et al., 2020). The fulfillment of professional commitment promoted consciousness of the nursing role and deepened the understanding of professional essence (Fan et al., 2020, Sheng et al., 2020, Sun et al., 2020, Tan et al., 2020, N Galehdar et al., 2020). As such, nurses expressed a sense of satisfaction for the opportunity to achieve their professional value (Sun et al., 2020, Zhang et al., 2020, Deliktas et al., 2021, Muz and Erdoğan, 2021, N Galehdar et al., 2020, Lee and Lee, 2020), and a sense of pride for their contributions to disease control (Jia et al., 2021, Sheng et al., 2020, Sun et al., 2020, Zhang et al., 2020, Muz and Erdoğan, 2021, Lee and Lee, 2020, Goh et al., 2020, Bennett et al., 2020). Furthermore, nurses reported that they felt valued by society which indicated that the social identity of nurses had increased (Deliktas et al., 2021, Sethi et al., 2020), and the prestige and position of the nursing profession had been enriched (Deliktas et al., 2021, Muz and Erdoğan, 2021, N Galehdar et al., 2020).

### 3.5 A call for future help and support

Since nurses directly experienced the drawbacks of insufficient resources and support, they proposed expectations for the provision of knowledge, training (Arnetz et al., 2020, Hou et al., 2020, Q Liu et al., 2020, YE Liu et al., 2020, Coşkun and Günay, 2021, Lee and Lee, 2020), and protective materials (Arnetz et al., 2020, Góes et al., 2020, Tan et al., 2020, N Galehdar et al., 2020, Sethi et al., 2020, Vindrola-Padros et al., 2020, Okediran et al., 2020), including PPE and testing. Moreover, studies reported that nurses expressed a strong need for psychological resources from health-care organisations and leaders (Arnetz et al., 2020, Gao et al., 2020, Góes et al., 2020, Kackin et al., 2020, Kalateh et al., 2020, Tan et al., 2020, Lee and Lee, 2020, Okediran et al., 2020). Interestingly, nurses in two studies reported that they received adequate psychological support from the hospital (Q Liu et al., 2020, Goh et al., 2020), yet many other studies pointed to the contrary and that nurses experienced multiple psychological symptoms due to lack of psychological interventions (Kackin et al., 2020, Tan et al., 2020, Deliktas et al., 2021, Lee and Lee, 2020, Goh et al., 2020). The nurses were disappointed by their respective organisations’ leadership during the pandemic due to unreasonable arrangements and scheduling (Arnetz et al., 2020, Gao et al., 2020, Góes et al., 2020, Kalateh et al., 2020), and felt that the managers neglected their opinions (Gao et al., 2020, Góes et al., 2020). Accordingly, nurses hoped that leaders could arrange work more appropriately in consideration of their physical and mental well-being (Gao et al., 2020), thereby increasing the trust in the workplace and improving the efficiency of nursing work (Arnetz et al., 2020, Kalateh et al., 2020). In addition, nurses put forward the need for social support (Eftekhar et al., 2020, Hou et al., 2020, Sheng et al., 2020, Muz and Erdoğan, 2021, N Galehdar et al., 2020, Fernández-Castillo et al., 2021, Lee and Lee, 2020, Okediran et al., 2020). Some nurses expressed expectations for appropriate and timely financial allowance or reimbursement for their work during the pandemic (Eftekhar et al., 2020, Sheng et al., 2020, Muz and Erdoğan, 2021, N Galehdar et al., 2020, Lee and Lee, 2020, Okediran et al., 2020), employee rights (Muz and Erdoğan, 2021), and logistical support from the hospital (Fernández-Castillo et al., 2021, Okediran et al., 2020).

During later stages of the pandemic, when nurses experienced pandemic fatigue, a study showed that the early fear, anxiety, and helplessness of nurses appeared to have been reactivated due to resurgences in the ongoing pandemic (Eftekhar et al., 2020). The possibility of care for COVID-19 patients becoming a long-term work requirement was a concern in many studies, thus not only compelling nurses to make psychological preparations, but also suggesting the need for adequate long-term material and psychosocial preparations to support nurses (Eftekhar et al., 2020, Lee and Lee, 2020).

### 3.6 Confidence in cumulative evidence

The evidence was assessed using the Confidence in the Evidence from Reviews of Qualitative research (CERQual) Approach (Lewin et al., 2015). CERQual provides a systematic and transparent framework for assessing confidence in each review finding in terms of methodological limitations, relevance, coherence, and adequacy of data (Lewin et al., 2018). The levels of confidence in each individual review finding can be reported as high, moderate, low and very low (Lewin et al., 2018). Based on CERQual assessment, the confidence in two findings was high and in one finding was moderate (Table 5).

**Table 5:** CERQual Qualitative Evidence Assessment.

Objective: To explore the psychosocial experiences of frontline nurses working in hospital-based settings during the COVID-19 pandemic.							
Included studies: Twenty-eight studies (Arnetz et al., 2020, Eftekhar et al., 2020, Fan et al., 2020, N Galehdar et al., 2020, Gao et al., 2020, Góes et al., 2020, Hou et al., 2020, Jia et al., 2021, Kackin et al., 2020, Kalateh et al., 2020, Q Liu et al., 2020, YE Liu et al., 2020, Sheng et al., 2020, Sun et al., 2020, Tan et al., 2020, Zhang et al., 2020, Deliktas et al., 2021, Muz and Erdoğan, 2021, Coşkun and Günay, 2021, N Galehdar et al., 2020, Sethi et al., 2020, Fernández-Castillo et al., 2021, Ohta et al., 2020, Lee and Lee, 2020, Goh et al., 2020, Bennett et al., 2020, Vindrola-Padros et al., 2020, Okediran et al., 2020)							
Review Finding	Studies Contributing to the Review Finding	Assessment of Methodological Limitations	Assessment of Relevance	Assessment of Coherence	Assessment of Adequacy	Overall CERQual Assessment of Confidence	Explanation of Judgement
Frontline nurses experienced fear of infection and uncertainty during the COVID-19 pandemic.	Studies Arnetz et al., 2020, Eftekhar et al., 2020; Galehdar et al., 2020a, Gao et al., 2020, Góes et al., 2020; Kackin et al., 2020, Kalateh et al., 2020, Liu et al., 2020a, Liu et al., 2020b, Sheng et al., 2020, Sun et al., 2020, Tan et al., 2020, Zhang et al., 2020, Deliktas et al., 2021, Muz and Erdoğan, 2021, Coşkun and Günay, 2021, Galehdar et al., 2020b, Sethi et al., 2020, Fernández-Castillo et al., 2021, Ohta et al., 2020, Lee and Lee, 2020, Goh et al., 2020, Bennett et al., 2020	Minor methodological limitations (23 studies with minor methodological limitations)	Minor concerns about relevance (the studies were from 11 countries)	Minor concerns about coherence (data reasonably consistent within and across all studies)	Minor concerns about adequacy (11 studies together offered moderately thick data overall)	**High confidence**	This finding was graded as high confidence because of minor concerns regarding methodological limitations, relevance, coherence, and adequacy.
The unfamiliarity in the workplace and psychological unpreparedness were the main occupational stressors that caused nurses psychological distress and negative physical impacts.	Studies Arnetz et al., 2020, Eftekhar et al., 2020, Fan et al., 2020, Galehdar et al., 2020a, Gao et al., 2020, Góes et al., 2020; Kackin et al., 2020, Kalateh et al., 2020, Liu et al., 2020a, Liu et al., 2020b, Sheng et al., 2020, Sun et al., 2020, Tan et al., 2020, Zhang et al., 2020, Deliktas et al., 2021, Muz and Erdoğan, 2021, Coşkun and Günay, 2021, Galehdar et al., 2020b, Sethi et al., 2020; Ohta et al., 2020; Lee and Lee, 2020, Bennett et al., 2020, Okediran et al., 2020	Minor methodological limitations (23 studies had minor methodological limitations)	Minor concerns about relevance (the studies were from 10 countries)	Minor concerns about coherence (data consistent within and across all studies)	Minor concerns about adequacy (10 studies together offered moderately thick data overall)	**High confidence**	This finding was graded as high confidence because of minor concerns regarding methodological limitations, relevance, coherence, and adequacy.
Nurses’ coping strategies combined with external supports given to nurses contributed to improved coping abilities to stress and thus increased professional identity	Studies Eftekhar et al., 2020, Fan et al., 2020, Galehdar et al., 2020a, Gao et al., 2020; Hou et al., 2020, Jia et al., 2021, Kackin et al., 2020; Liu et al., 2020a, Liu et al., 2020b, Sheng et al., 2020, Sun et al., 2020; Zhang et al., 2020, Deliktas et al., 2021, Muz and Erdoğan, 2021, Coşkun and Günay, 2021, Galehdar et al., 2020b, Sethi et al., 2020, Fernández-Castillo et al., 2021, Ohta et al., 2020, Lee and Lee, 2020, Goh et al., 2020, Bennett et al., 2020, Vindrola-Padros et al., 2020, Okediran et al., 2020	Minor methodological limitations (24 studies had minor methodological limitations)	Minor concerns about relevance (the studies were from 10 countries)	Moderate concerns about coherence (some concern about the fit between the data from a few primary studies)	Minor concerns about adequacy (10 studies together offered moderately thick data overall)	**Moderate confidence**	This finding was graded as moderate confidence because of minor concerns regarding methodological limitations, relevance, and adequacy; and moderated concerns regarding coherence.

## 4 Discussion

### 4.1 Summary of evidence

This study systematically reviewed 28 qualitative studies to synthesize the psychosocial experiences of frontline nurses working in hospital-based settings during the COVID-19 pandemic. The main findings that will be discussed in this section indicated that frontline nurses experienced fear of infection and uncertainty during the COVID-19 pandemic. Further, unfamiliarity in the workplace and psychological unpreparedness were the main occupational stressors that caused nurses’ psychological distress and negative physical impacts. Moreover, nurses’ coping strategies combined with external support contributed to improved coping abilities in terms of stress management and a strengthened sense of professional competence in nurses.

During the COVID-19 pandemic, nurses experience fear, anxiety, and psychological distress due to the risk of infection, concerns about family members, and the uncertainty of the disease. Compared with evidence from previous pandemics, such as severe acute respiratory syndrome (SARS) and the Middle East respiratory syndrome (MERS) outbreak, it is consistent that nurses’ concerns about their own safety heightened their anxiety level (Lam and Hung, 2013, Holroyd and McNaught, 2008, Kang et al., 2018, Koh et al., 2012). The fears of the unknown, virus infection and transmission are the prominent stressors related to the pandemic (Maunder et al., 2004, Bai et al., 2004, Chua et al., 2004, Ornell et al., 2020, Chan et al., 2005, Shih et al., 2007, Wong et al., 2005, Khalid et al., 2016, Khee et al., 2004, Wu et al., 2009). Although frontline nurses try to isolate themselves, they are concerned about the safety of their family members (Lam and Hung, 2013, Koh et al., 2012, Wong et al., 2005, LoGiudice and Bartos, 2021, Ives et al., 2009). The previous evidence indicates that quarantining resulted in adverse effects on nurses’ mental health (Rossi et al., 2020), and social distancing deprives nurses of social support at work and added work-related stress (Halcomb et al., 2020, Labrague and de Los Santos, 2021). Social restrictions weaken nurses’ social relationships yet trigger stigma towards nurses (Kim, 2018- (Xiang et al., 2020). During the SARS outbreak, nurses who experienced social avoidance and stigma developed higher levels of PTSD (Maunder et al., 2004). Evidence suggested that accurate messages from public health authorities could reduce underlying stigma and fear that is cultivated through media (Shih et al., 2007, Sirois and Owens, 2021).

The findings reveal that nurses experienced an inevitable sense of a loss of control when facing the suffering of patients combined with the perceived risks in contaminated environments, though having a sense of control is vital for nurses to resist distress and anxiety (Gallagher et al., 2014, Taylor et al., 2020). Evidence indicates that healthcare professionals frequently met ethical challenges in difficult work conditions (Mert et al., 2020, Cerit and Dinç, 2013, Amiri et al., 2019). Insufficient knowledge and resources, uncertainty with duties and procedures, and communication problems are the major causes of ethical issues and moral distress felt by nurses, and it is consistently noted that nurses are unable to provide adequate care when facing these challenges (Dehghani et al., 2015, Mert et al., 2021, Silverman et al., 2021). On the other hand, taking care of patients is a professional commitment that needs to be fulfilled (Kang et al., 2018, Koh et al., 2012). Consistent with the evidence, the fulfillment of professional duty brought a sense of pride and accomplishment to nurses. However, nurses experience moral distress pertaining to the maintenance of professional obligations and a sense of powerlessness in providing adequate care in a challenging environment (Silverman et al., 2021). Evidence emphasised that working with a high risk of infection due to professional obligation while having to work with insufficient protective measures creates ethical dilemmas and moral distress for nurses during pandemics (Mert et al., 2021, Silverman et al., 2021).

To cope with stress, frontline nurses used adaptive coping strategies, such as active learning, and maladaptive coping strategies, such as self-blaming, all while highlighting the need for psychological management at the leadership and organisational levels. Evidence indicates that adaptive coping strategies could effectively alleviate or prevent stress (Wang et al., 2020), while maladaptive coping strategies might lead to higher levels of burnout and PTSD as long-term impacts in previous pandemics (Maunder et al., 2006, Nie et al., 2020). Coping styles and perceived social support are associated with individual management of stress (Mariani et al., 2020). Social support is unanimously indicated to lower the level of distress and improve nurses’ mental health (Spoorthy et al., 2020, Sirois and Owens, 2021, Taylor et al., 2020, Nie et al., 2020). Corroborating previous studies, organisational support diminishes the perceived fears and emotional exhaustion of nurses (Sirois and Owens, 2021, Ng and Sorensen, 2008), particularly since nurses rely on organisational initiatives and expect to receive clear and adequate knowledge about the pandemic in a consistent manner (Fernandez et al., 2020). There has been increasing evidence supporting that the COVID-19 pandemic could be understood as a traumatic event (Bridgland et al., 2021). Studies report that frontline nurses developed PTSD after SARS and MERS outbreaks (Chan and Huak, 2004, Lancee et al., 2008, Lee et al., 2018, Kim et al., 2018). Therefore, trauma care might be crucial for nurses’ psychological and mental well-being, and obtaining trauma-related knowledge can support nurses in coping with personal and occupational stressors (Eslami et al., 2015, Fowler and Wholeben, 2020).

### 4.2 Strengths and limitations

This systematic review focused on the voices of frontline nurses regarding their psychological, social, emotional experiences during the COVID-19 pandemic. The results provide insights into nurses' perspectives on the challenges in frontline work management and barriers to the delivery of care. The included studies were from 12 different countries from five continents. Hence multiple perspectives from a diversity of countries are represented in the results, speaking for transferability of the results across different countries. This review corroborates previous pandemic research, and the results of this review contribute to the knowledge base about nurses’ professional and personal stressors in frontline work during the COVID-19 pandemic, including nurses’ psychosocial experiences in coping with work, social relationships, and personal life.

However, this systematic review has several limitations, which should be taken into consideration in light of the results’ interpretation. Several limitations discussed below relate to the included studies’ own prerequisites and limitations, but also to the current review's delimitations and limited time frame. First, the response to the pandemic in different countries may lead to various protocols and policies, which may influence frontline nurses’ attitudes and work experiences. Second, the review did not take sociodemographic characteristics into consideration, though sociodemographic factors may have significant influence on frontline nurses’ psychological experiences during a pandemic (Maunder et al., 2004). Third, nurses’ work-related background information was not collected. A nurse's typical work position and department may influence transdisciplinary nurses’ work experiences in COVID-19 wards (Fan et al., 2020). Furthermore, frontline nurses can be found in additional contexts not represented in the current findings, limiting the results’ transferability to care contexts differing from the ones represented in the current review. Fourth, the review limited the language of published studies to those published in English. Studies published in other languages, and countries or regions where the COVID-19 pandemic was prevalent may have been excluded. Fifth, the citation search was limited to the database of WOS, which has a more limited number of journals than SCOPUS or Dimensions (Singh et al., 2021). It means that potentially relevant articles could have been overlooked. Lastly, the review only included peer-review studies published before February 2021. gray literature and pre-printed research which may contain relevant COVID-19 knowledge have been excluded.

### 4.3 Implications for nursing and health policy

This qualitative systematic review highlights the significance of frontline nurses’ experience of psychological, social and emotional distress during the COVID-19 pandemic. Maintaining the mental health of frontline nurses is crucial to the quality of care and control over the pandemic (Mo et al., 2020). It is suggested that policymakers, health-care organisations, nursing managers, and nursing leaders engage in supporting frontline nurses during the pandemic. Nursing leaders should not only pay attention to the challenges that frontline nurses have experienced in their delivery of care, but aim to guide nurses in using adaptive coping strategies to prevent negative effects on their mental well-being. Nursing managers should provide safe and healthy working conditions for frontline nurses. It may also be helpful to offer corresponding financial subsidies or rewards and ensure professional equality as a way to mobilise the motivation and enthusiasm of frontline nurses (Mo et al., 2020). There is an evident need for health-care organisations to provide necessary resources and support to mitigate psychological and moral distress in frontline nurses.

Psychological interventions should be implementable and readily accessible for all frontline nurses to help them cope with psychological and emotional distress (Fernandez et al., 2020). Multidimensional social support is essential for frontline nurses in managing stress and maintaining mental well-being. Education aimed at nurses may be critical in lessening social stigma (Fernandez et al., 2020). Policy-makers should address the barriers that create ethical challenges for frontline nurses and consider multifaceted support to optimize working conditions (Mert et al., 2021), in order to help deal with wide psychosocial issues and promote nurses’ professional identity.

## Conclusion

Nurses working frontline during the COVID-19 pandemic have experienced psychological, social, and emotional distress in coping with work, social relationships, and their personal life. COVID-19 generates multiple challenges to the frontline nursing practice. The results speak of a need for psychological and social support for frontline nurses to cope with stress and maintain mental well-being, which may subsequently affect the outcomes and efficiency of nursing care. It is vital for nursing leaders, nursing managers, health-care organisations, and policy-makers to provide multifaceted support to increase professional satisfaction and ensure sustainability of the nursing workforce. Future research is needed to explore long-term psychosocial experiences of COVID-19 frontline nurses. Such evidence may serve as a guide for nurses’ mental health management in response to future public health emergencies.

## Others

• Data availability statement: publicly available

• No funding to declare

## CRediT authorship contribution statement

**Hongxuan Xu:** Conceptualization, Methodology, Formal analysis, Writing – original draft, Writing – review & editing, Project administration. **Sigrid Stjernswärd:** Conceptualization, Methodology, Formal analysis, Writing – original draft, Writing – review & editing. **Stinne Glasdam:** Conceptualization, Methodology, Formal analysis, Writing – original draft, Writing – review & editing.

## Declaration of Competing Interest

No conflict of interest has been declared by the authors.
